# Frontotemporal dementia: does structural MRI-based clustering match clinical syndromes?

**DOI:** 10.3389/fnins.2026.1771092

**Published:** 2026-05-05

**Authors:** Neha Singh-Reilly, Irene Sintini, Farwa Ali, Joseph R. Duffy, Heather M. Clark, Rene L. Utianski, Gabriela Meade, Mary M. Machulda, Ryota Satoh, Val J. Lowe, Keith A. Josephs, Jennifer L. Whitwell

**Affiliations:** 1 Department of Radiology, Mayo Clinic, Rochester, MN, United States; 2 Department of Neurology, Mayo Clinic, Rochester, MN, United States; 3 Department of Psychiatry & Psychology, Mayo Clinic, Rochester, MN, United States

**Keywords:** atrophy, clinical syndromes, data-driven clustering analysis, frontotemporal dementia, MRI

## Abstract

**Background:**

Frontotemporal dementia is an umbrella term that encompasses several clinical syndromes with impaired behavioral, language, and motor functions. These syndromes show considerable overlap in clinical features and imaging patterns. Therefore, there is a need to investigate the syndromic heterogeneity in FTD using unbiased data-driven approaches.

**Methods:**

We used data-driven clustering analysis of structural magnetic resonance imaging (MRI) data on 400 patients with clinical FTD diagnoses [behavioral variant of frontotemporal dementia (bvFTD), semantic variant of primary progressive aphasia (svPPA), right temporal variant of frontotemporal dementia (rtvFTD), apraxia of speech with agrammatic aphasia (AOS-PAA), primary progressive apraxia of speech (PPAOS), progressive supranuclear palsy (PSP), corticobasal syndrome (CBS) and primary progressive aphasia who did not fit into the other diagnostic categories (PPA-other)]. MR images were w-scored relative to cognitively unimpaired individuals, and principal component analysis was performed. A clustering ensemble approach, including hierarchical algorithms, was applied to the MR-based principal components, and imaging and clinical characteristics of the clusters were investigated. Various numbers of clusters (*K* = 2, 3, or 4) were evaluated.

**Results:**

The *K* = 3 solution offered the most clinically meaningful separation of FTD syndromes. The first cluster captured mostly frontal MRI abnormalities related to the speech, language and behavioral clinical dimensions, including patients with AOS-PAA, PPAOS, PPA-other, and bvFTD. The second cluster captured mostly temporal abnormalities and included mainly patients with svPPA and rtvFTD, but also bvFTD, AOS-PAA, and PPA-other. The third cluster captured cortical and subcortical atrophy, particularly in the midbrain, and included atypical Parkinsonian syndromes, with all PSP and CBS patients captured in this cluster, as well as most PPAOS patients. Considerable overlap of clinical syndromes was noted across these clusters, whereby patients with AOS-PAA, svPPA, PPA-other, and bvFTD were captured in more than one cluster.

**Discussion:**

Our findings highlight heterogeneity in FTD, which mainly exists along three axes: speech, language and behavioral deficits reflecting frontal atrophy, language deficits reflecting temporal atrophy, and motor and motor speech deficits reflecting mostly midbrain and subcortical atrophy, with cortical involvement.

## 1 Introduction

Frontotemporal dementia (FTD) is a neurodegenerative disorder that subsumes a range of clinical syndromes that affect the frontal and temporal lobes, and are associated with a frontotemporal lobar degeneration (FTLD) pathology (Josephs, 2007). The original diagnostic criteria for FTD (Neary et al., 1998) recognized three clinical syndromes, including the behavioral variant of FTD (bvFTD), characterized by changes in behavioral and personality, and impairment in executive function (Rascovsky et al., 2011); semantic dementia characterized by loss of semantic knowledge (Hodges et al., 1992), and progressive non-fluent aphasia (PNFA), characterized by non-fluent spontaneous speech output. Since then, the semantic dementia and PNFA categories have been redefined. It has been recognized that semantic dementia can predominantly target either the left or right hemisphere, with left-sided cases presenting with anomia and loss of word meaning and captured under the term semantic variant of primary progressive aphasia (svPPA) (Gorno-Tempini et al., 2011). Right predominant semantic dementia cases present with behavioral abnormalities, difficulty recognizing faces and anomia, and have been referred to as right temporal variant of FTD (rtvFTD) (Ulugut Erkoyun et al., 2020; Josephs et al., 2009). It has been recognized that the PNFA category includes patients with agrammatism in written and spoken language and/or the motor speech disorder of apraxia of speech (AOS) in which patients have trouble in the planning and programming of speech. Patients can present with both AOS and progressive agrammatic aphasia (PAA) and we will refer to these patients as AOS-PAA (Ogar et al., 2007), although they could also be captured under the diagnosis of agrammatic/non-fluent variant of primary progressive aphasia (nfvPPA) (Gorno-Tempini et al., 2011). Patients that present with an isolated AOS, without agrammatic aphasia, are diagnosed as primary progressive apraxia of speech (PPAOS) (Josephs et al., 2012). The PNFA category was also found to include patients with the logopenic variant of primary progressive aphasia (lvPPA) who present with anomia, difficulty with sentence repetition and phonological errors (Gorno-Tempini et al., 2011). It was found that most lvPPA patients have underlying Alzheimer’s disease (AD) (Whitwell et al., 2015; Spinelli et al., 2017; Santos-Santos et al., 2018), although some had FTLD pathology (Whitwell et al., 2015; Josephs et al., 2014b). In addition to these FTD syndromes, the atypical parkinsonian syndromes of progressive supranuclear palsy (PSP), characterized by ocular motor slowing and frequent falls (Höglinger et al., 2017) and corticobasal syndrome (CBS), characterized by asymmetric rigidity, myoclonus, cortical sensory loss and ideomotor apraxia (Armstrong et al., 2013), have been considered as FTD syndromes since they can also affect the frontal lobes and are associated with FTLD pathology.

Although clinical assessment is critical in the diagnostic workup of these syndromes, neuroimaging is an equally important variable, as each syndrome is associated with structural changes in specific brain regions, with the frontotemporal cortices and subcortical regions showing visible signs of neuronal loss on magnetic resonance imaging (MRI) and matching post-mortem pathological findings (Whitwell et al., 2009; Whitwell et al., 2005). Patterns of atrophy within these regions varies across syndrome, with bvFTD predominantly affecting the prefrontal and anterior temporal cortices (Eldaief et al., 2023); rtvFTD affecting the anterior temporal lobes, particularly in the right hemisphere (Ulugut Erkoyun et al., 2020); svPPA affecting the anterior temporal lobes, particularly in the left hemisphere (Gorno-Tempini et al., 2004); PAA affecting the prefrontal cortex, including Broca’s area (Botha et al., 2015); AOS-PAA affecting the medial and lateral premotor cortex and Broca’s area (Botha et al., 2015); PPAOS affecting the medial and lateral premotor cortex (Josephs et al., 2014a) and lvPPA affecting left lateral temporal and inferior parietal regions (Whitwell et al., 2015; Gorno-Tempini et al., 2004). The atypical Parkinsonian syndromes show extensive subcortical atrophy in the midbrain and basal ganglia, but also frontal atrophy, particularly in the supplementary motor area (Whitwell et al., 2020; Whitwell et al., 2010). Overlap is observed across the FTD syndromes, making differentiation among syndromes challenging (Murley et al., 2020).

In this study, we aimed to utilize data-driven clustering methods applied to structural MRI data from a large cohort of patients with FTD to determine whether structural MRI-based clusters would capture the FTD clinical syndromes and determine how these clusters relate to clinical characteristics in FTD. These analyses were intended to further our understanding of heterogeneity in FTD and determine the degree to which structural MRI can be utilized to aid the differential diagnosis of the different FTD syndromes. Employing unsupervised clustering approaches that do not rely on clinical diagnosis offer an unbiased approach to understand biological heterogeneity in FTD (Spinelli et al., 2025; Inguanzo et al., 2023; Corriveau-Lecavalier et al., 2024; Dong et al., 2017). For this study, we selected hierarchical clustering, which is a classical clustering technique that has been extensively applied to neuroimaging of neurodegenerative diseases (Inguanzo et al., 2023; Tan et al., 2022; Inguanzo et al., 2021; Uribe et al., 2018). We hypothesized that data-driven clusters would group structural MRI-based covariance patterns from principal component analysis (PCA) in a way that reflects the main domain of impairment of the patients, with clear distinction between motor and semantic domains, confirming the utility of structural MRI in capturing disease-related heterogeneity across clinical syndrome with both distinct and overlapping features and of principal component analysis as a data-reduction method in neuroimaging.

## 2 Methods

### 2.1 Patients

Four hundred patients with a clinical FTD diagnosis were recruited by the Neurodegenerative Research Group (NRG) from the Department of Neurology, Mayo Clinic, Rochester, MN, between 2009 and 2024. The cohort consisted of patients that met clinical criteria for bvFTD (*n* = 12) (Rascovsky et al., 2011), svPPA (*n* = 28) (Gorno-Tempini et al., 2011), rtvFTD (*n* = 11) (Miller et al., 1993), AOS-PAA (*n* = 70), PPAOS (*n* = 53) (Josephs et al., 2012), PSP (*n* = 185) (Höglinger et al., 2017), and CBS (*n* = 23) (Armstrong et al., 2013). In addition, we had 18 patients who met criteria for PPA (Gorno-Tempini et al., 2011) who did not fit into the other diagnostic categories and hence were labelled PPA-other. The PPA-other group consists of 10 patients with PAA, four patients with lvPPA who were biomarker-confirmed not to have AD, and four patients with PPA who could not be classified into any specific syndrome. Patients with CBS were required to be either tau-negative or amyloid-negative on positron emission tomography (PET) and patients with PPA-other (logopenic variant) were required to be amyloid-negative to rule out AD (Jack et al., 2017). All patients underwent neurological evaluations by a behavioral neurologist (KAJ), neuropsychological testing (MMM), a speech and language battery performed by one of three board certified speech-language pathologists (JRD, HMC or RLU) and completed a structural MRI. One hundred and nine cognitively unimpaired individuals (age: 62 ± 8, MoCA: 27 ± 2, 32 females, 96 on Siemens MRI scanner) were also recruited by the NRG between 2016 and 2024 and completed the same imaging protocols. Clinical diagnosis was rendered blinded to imaging results.

### 2.2 Clinical testing

The neurological and neuropsychological evaluations included the Montreal Cognitive Assessment (MoCA) to assess general cognitive function (Nasreddine et al., 2005), Movement Disorders Society sponsored revision of the Unified Parkinson’s disease rating scale III (MDS-UPDRS III) to assess parkinsonism (Martinez-Martin et al., 1994), PSP Rating Scale to assess the severity of PSP clinical features (Golbe and Ohman-Strickland, 2007), the Western Aphasia Battery ideomotor apraxia (WAB praxis) subtest to assess for ideomotor apraxia (Shewan and Kertesz, 1980) and Frontal Assessment Battery (FAB) to assess executive functions (Dubois et al., 2000). Language assessment included the Western Aphasia Battery-Revised (WAB) (Kertesz, 2007), specifically the animal fluency subscore to assess category fluency performance (Scheffel et al., 2021), the Aphasia Quotient (WAB AQ) subtest to measure global language ability and aphasia severity, and the repetition subset of the WAB to assess sentence repetition (Kertesz, 2007), the letter fluency sum to assess lexical fluency performance (Scheffel et al., 2021), the word–word version of the Pyramids and Palm Trees (PPT) to assess word knowledge (Howard and Patterson, 1992), the Apraxia of Speech Rating Scale (ASRS) to rate the severity of apraxia of speech characteristics (Duffy et al., 2023) and the 15-item Boston Naming Test-short form (BNT-SF) to assess confrontation naming (Lansing et al., 1999).

### 2.3 Image acquisition and processing

All patients were scanned using a 3 T volumetric MRI on GE (GE Healthcare, Milwaukee) (*n* = 210) or Siemens (Magnetom Prisma, Siemens Healthineers) (*n* = 190) scanners at Mayo Clinic, Rochester, MN. The scan included a 3D magnetization-prepared rapid gradient echo (MPRAGE) sequence (TR/TE/TI, 2300/3/900 ms; flip angle 8°, 26-cm field of view; 256 × 256 in-plane matrix with a phase field of view of 0.94, and slice thickness of 1.2 mm) (Satoh et al., 2023). All scans met quality control measures, with a quality control analyst rating of 1–3 (on a scale of 1 to 4, with 1 being high quality and 4 being lowest quality), indicating that the scans had a good signal to noise ratio and no significant blurring, motion, ringing, ghosting, flow or linear artifacts, susceptibility, distortion, wrap or head coverage issues. All MPRAGE scans were segmented into gray matter and white matter bias-field corrected using Unified Segmentation (Ashburner and Friston, 2005) in SPM12 (Wellcome Trust Centre for Neuroimaging, London, UK), with Mayo Clinic Adult Lifespan Template (MCALT)^1^ tissue priors and settings (Schwarz et al., 2017). Images for each patient were subsequently spatially normalized to the MCALT template and smoothed with an 8-mm full width at half maximum kernel for the voxel-based analyses.

### 2.4 Clustering analysis

PCA was performed on *w*-scored gray and white-matter segmentations. We applied the Kaiser–Meyer–Olkin (KMO) criterion to verify that PCA could reduce the dimensionality of the data in a meaningful way. The structural MR images were *w*-scored before applying PCA to control for the influence of normal aging, sex, total intracranial volume (TIV) and scanner manufacturer (La Joie et al., 2012). *W*-scoring was performed at the voxel-level. To create *w*-scored images for the FTD patients, we derived a linear regression relationship between the gray and white matter segmentation maps of the cognitively unimpaired participants and the covariates of interest (age, sex, scanner manufacturer, total intracranial volume) using SPM. The linear regression coefficients were then applied to the FTD participants characteristics to predict the ‘healthy version’ of their gray and white matter segmentation maps. The *w*-scored images were obtained as the difference between the real image and the predicted ‘healthy’ image, divided by the standard deviation of the residuals from the linear regression on the cognitively unimpaired. Clustering was applied to the similarity matrix obtained from the first 12 patient–specific principal component scores, each of which explained at least 1% of the data variability, using Manhattan distance metrics. The diverse clustering ensemble algorithm *dice* in the package *diceR* (R version 4.4.3) was employed, including hierarchical clustering algorithms (namely: hc, DIANA) (diceR: vignettes/overview.Rmd). The *dice* function runs the clustering algorithms of choice on subsamples which include 80% of the full cohort for *n* = 10 times. To further ensure clusters stability, we employed a leave-one-out (LOO) analysis and compared the cluster assignments obtained leaving one participant out to the ones obtained on the full cohort with the Rand index and adjusted Rand index (Hubert and Arabie, 1985). The clustering algorithm was run with the number of clusters (*K*) equal to 2, 3, or 4. The Calinski–Harabasz (CH) index was employed to evaluate the separations of the clusters. The clinical characteristics of the patients within each cluster were compared using the Kruskal–Wallis test. The atrophy patterns of each cluster were evaluated by comparing the gray and white matter MRI segmentations of the members of each cluster to the cognitively unimpaired cohort using SPM, covarying for age, sex, and scanner manufacturer. Similarly, the atrophy patterns of the clinical syndromes within each cluster were investigated. To rule out biases introduced by linearly regressing out covariates with *w*-scores obtained relative to a cohort of cognitively unimpaired individuals which was not perfectly balanced, clustering analysis was also performed on the principal components of the raw gray and white matter segmentation maps of the FTD patients. Analyses were run in R version 4.4.3.

## 3 Results

### 3.1 Patient demographics

The demographic and clinical features of the cohorts are shown in Table 1. Syndromes did not differ in sex and education. There were significant differences in age and disease duration, with PSP patients being the oldest and rtvFTD patients having the longest disease duration. The bvFTD group was the most impaired on the MoCA. The PSP and CBS groups performed the worst on the MDS-UPDRS III and the PSP rating scale, while the CBS group performed the worst on the WAB praxis. The AOS-PAA and PPAOS groups had the worse AOS severity; the PPA-other group had the lowest on naming and fluency measures; and the svPPA group performed the worst on word knowledge, fluency, and aphasia measures.

**Table 1:** Demographics and clinical characteristics of patients.

Characteristics	bvFTD *n* = 12	svPPA *n* = 28	rtvFTD *n* = 11	AOS-PAA *n* = 70	PPAOS *n* = 53	PPA-other *n* = 18	PSP *n* = 185	CBS *n* = 23	*p* value
Demographics									
Age (yrs)	66 (61.5, 72.7)	68.2 (59.7, 71.6)	64.2 (60.9, 69.3)	69 (62.9, 73.4)	72.7 (63.1, 77.3)	65.8 (62.8, 72.5)	70.4, (65.9, 75.4)	65.4, (59.8, 68.5)	**0.009**
Disease duration (yrs)	3.6 (2.3, 4)	4.3 (3, 5.8)	4.7 (4.4, 5.1)	3.8 (2.6, 5.6)	3.3 (2.1, 5.2)	2.1 (2, 3.1)	3.7, (2.5, 5.2)	2.8, (1.7, 3.4)	**0.011**
Male (*n*, %)	7, 58%	12, 43%	5, 45%	34, 49%	25, 47%	6, 33%	97, 52%	12, 52%	0.816
Education (yrs)	16 (14, 16)	16 (13, 16)	16 (15, 18)	15 (12, 16)	16 (14, 18)	15 (12, 16)	14, (12, 16)	15, (12, 16)	0.058
Clinical tests									
MoCA (30)	16 (14.5, 22)	19 (14, 21.5)	22.5 (18.2, 24)	21 (17, 25)	27 (25, 28)	22 (18, 24)	23 (19, 26)	23 (21, 24)	**<0.001**
MDS-UPDRS III	10 (2, 30.5)	0 (0, 2)	2 (0, 3.5)	17 (10, 25)	12 (5, 22)	3.5 (1, 7.2)	41 (30.5, 54.5)	29 (22.8, 40.5)	**<0.01**
PSP rating scale (100)	7 (4.5, 18.5)	5 (3, 6.5)	2 (1, 3)	18 (10, 28.5)	8 (4, 17)	6 (2.5, 10.2)^a^	37 (28.5, 46)	24 (20, 27.5)	**<0.001**
ASRS total v3 (52)	5 (2, 6)	0 (0, 1)	0 (0, 0)	22 (12, 29)	16 (11.5, 23)	1 (0, 3)	14 (11, 16)	15 (13, 16.5)	**<0.001**
FAB (18)	13 (8, 14.2)	15 (12.8, 16.2)	16 (15.5, 18)	13 (10, 16)	17 (15, 17)	14 (10, 16)	4 (2, 6.8)	20 (15, 26)	**<0.001**
BNT (15)	12 (8, 12)^a^	0 (0, 2)^a^	10.5 (10.2, 10.8)^a^	12 (10.5, 14)	14 (13, 15)	10 (6.5, 13)	14 (12, 14)	13 (11.8, 14)	**<0.001**
Letter fluency sum	11 (10, 11)	17.5 (12.2, 27.2)	21 (19, 30)	10 (6, 19)	20 (12.8, 31.2)	13 (6.5, 23.5)	5.5 (3.8, 7)^a^	9 (3.5, 17)^a^	**<0.001**
WAB praxis (60)	59 (47, 60)	57.5 (48, 59.2)	60 (59, 60)	52 (45, 57)	58 (55, 59.2)	56 (53.8, 58.2)	50 (37, 52)^a^	51 (49, 55)	**<0.001**
WAB AQ (100)	90.3 (87.2, 93.3)	80.4 (64.4, 91.7)	91.2 (87.6, 94.2)	85.2 (77.5, 92.6)	97.8 (96.6, 99.2)	89.3 (79.3, 92.7)	97 (93.6, 98.1)^a^	94.6 (92.5, 96.5)	**<0.001**
WAB repetition (10)	9.2 (9, 9.8)	8.9 (7.8, 9.3)	9.6 (9.2, 9.7)	8.8 (7.8, 9.5)	9.7 (9.4, 9.9)	9.1 (8.2, 9.6)	9.6 (9.4, 10)^a^	9 (8.6, 9.5)^a^	**<0.001**
WAB animal fluency	10 (9, 12)	7 (3, 9)	8 (6.5, 10)	11 (7, 14)	16 (14.2, 20)	9 (7, 12)	12 (7.5, 14)^a^	14.5 (11.2, 15)	**<0.001**
PPT word-word (52)	47.5 (46.2, 48.8)	39 (32, 43.5)	38.5 (34.2, 41.5)	49 (48, 50)	50 (49.2, 51)	48 (46, 51)	49 (49, 49)^a^	47 (47, 47)^a^	**<0.001**

Demographic and clinical data are reported as median (25th, 75th quartiles), number and percentage. *p*-values are from Kruskal–Wallis and Fisher’s exact tests. PSP: progressive supranuclear palsy. rtvFTD: right temporal variant of frontotemporal dementia. svPPA: semantic variant of primary progressive aphasia. AOS-PAA: apraxia of speech - primary agrammatic PPA. PPAOS: primary progressive apraxia of speech. bvFTD: behavioral variant of frontotemporal dementia. CBS: corticobasal syndrome. MoCA: Montreal cognitive assessment. MDS-UPDRS: movement disorder society-sponsored revision of the unified Parkinson’s disease rating scale. ASRS: apraxia of speech rating scale. FAB: frontal assessment battery. BNT: Boston naming test. WAB AQ: western aphasia battery aphasia quotient. PPT: pyramid and palm trees test.

a Score available for less than 50% of participants. Significant *p* values are in bold.

### 3.2 Clustering analysis

**Figure 1:**
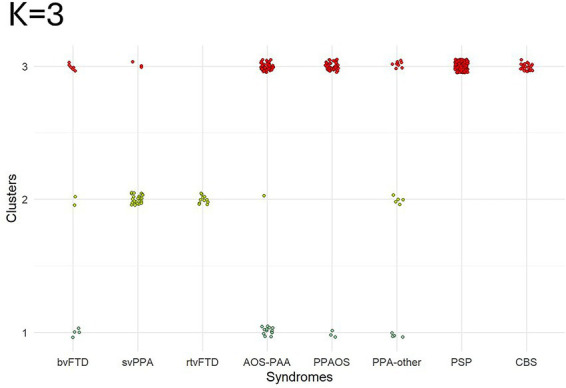
Syndrome distribution within each cluster (*K* = 3). Each one of the 400 patients was assigned to one of the three clusters. Cluster 1 included bvFTD, PPAOS, AOS-PAA, and PPA-other patients. Cluster 2 included bvFTD, svPPA, rtvFTD, AOS-PAA, and PPA-other patients. Cluster 3 included patients of all syndromes except rtvFTD.

The KMO criterion applied to the PCA input matrix (*w*-scored voxel-based gray and white matter probabilities across all participants) yielded an index of 0.96, which indicates that the data were suitable for PCA. The highest CH index was observed with *K* = 2 clusters (CH = 24.4), whereas the *K* = 3 solution yielded a lower index (CH = 21.1). The *K* = 4 solution led to the lowest index (CH = 20.3). Even though the two-cluster solution had the best cluster separation (i.e., highest CH index), the three-cluster solution provided the most clinically meaningful separation, and its results were used as the primary analysis for this study (Figure 1). LOO analysis yielded clusters that were similar to the ones obtained on the full cohort with *K* = 3 [Rand index: 0.90 (0.88, 0.91), adjusted Rand index: 0.75 (0.70, 0.78)].

**Figure 2:**
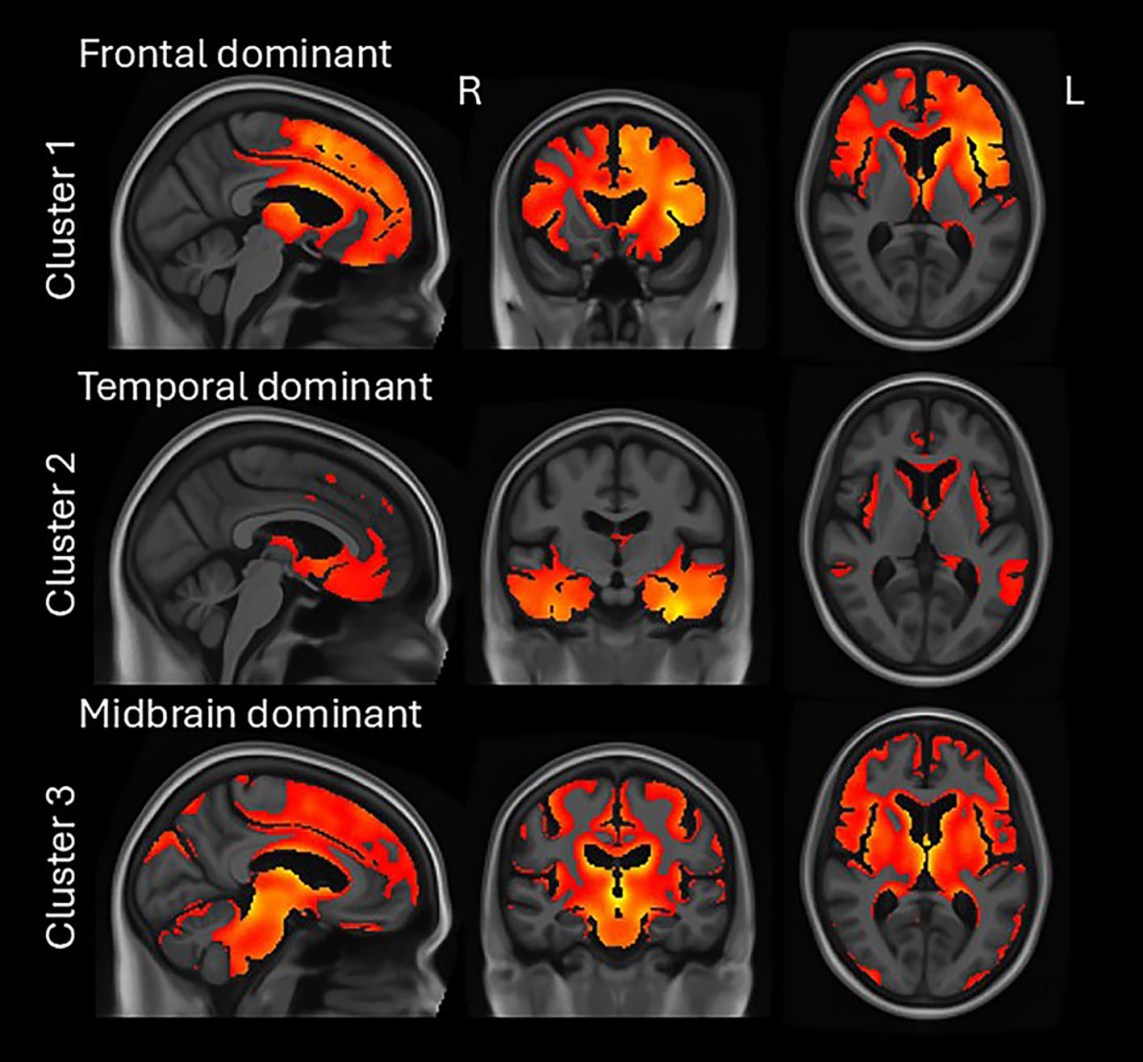
Atrophy patterns in each cluster. SPM maps of atrophy (gray and white matter segmentation) in each cluster relative to cognitively unimpaired individuals, at *p* < 0.05 with FWE correction covarying for age, sex and scanner manufacturer.

**Figure 3:**
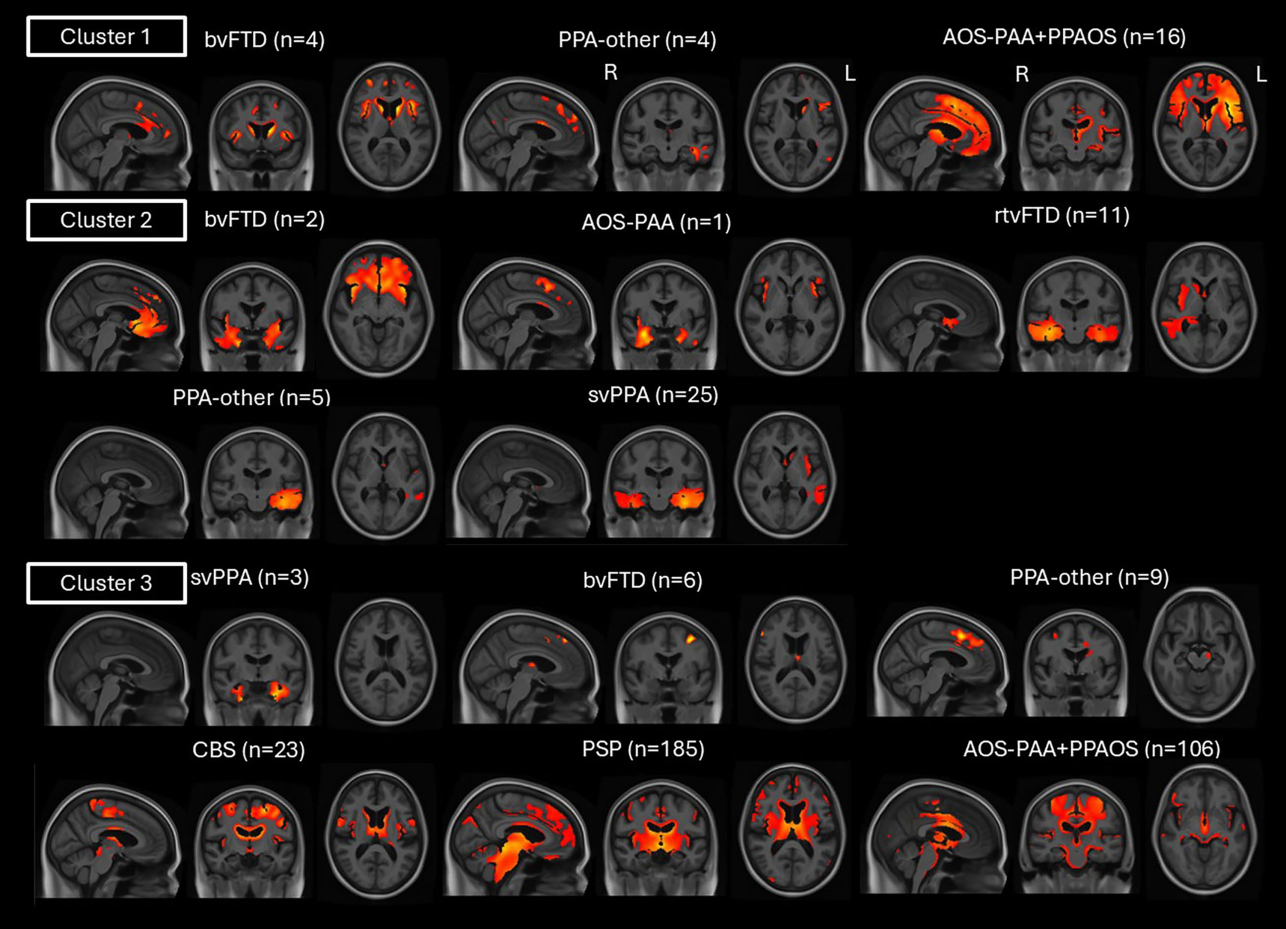
Atrophy patterns of each syndrome within a cluster. SPM maps of atrophy (gray and white matter segmentation) in each syndrome in cluster 1, cluster 2, and cluster 3 relative to cognitively unimpaired individuals, at *p* < 0.05 with FWE correction covarying for age, sex and scanner manufacturer. AOS-PAA and PPAOS were combined in the same group.

Cluster 1 (*n* = 24) mainly included patients with AOS (*n* = 16, 67%), i.e., AOS-PAA and PPAOS, followed by small subsets of PPA-other (*n* = 4, 16.5%) and bvFTD patients (*n* = 4, 16.5%) (Figure 1). On clinical testing, this cluster performed worse than the other two on FAB, Letter Fluency Sum and WAB repetition. Additionally, it performed worse than cluster 2 on MDS-UPDRS III, PSP Rating Scale, ASRS and WAB praxis, and worse than cluster 3 on MoCA, BNT, WAB AQ, and WAB animal fluency (Table 2). Gray matter volume loss was observed throughout the frontal lobe, basal ganglia and thalamus compared to cognitively unimpaired individuals (Figure 2). The AOS-PAA and PPAOS patients assigned to cluster 1 showed widespread volume loss in the frontal lobe, including the supplementary motor area, as well as the insula, cingulum, basal ganglia, and thalamus. The PPA-other patients showed left-sided volume loss in the inferior temporal and superior frontal regions, including also the cingulum and caudate. The bvFTD patients showed volume loss in the frontal lobe, including the supplementary motor area, and the insula, cingulum and basal ganglia (Figure 3).

**Table 2:** Demographic and clinical characteristics of the patients within each cluster.

Characteristics	Cluster 1 *N* = 24	Cluster 2 *N* = 44	Cluster 3 *N* = 332	*p* value overall	*p* value 1 vs 2	*p* value 1 vs 3	*p* value 2 vs 3
Age	63.5 (57, 67.7)	64.3 (58.5, 69.7)	70.4 (65.3, 75.5)	< 0.001	ns	< 0.001	< 0.001
Disease duration	3.1 (2.6, 4.6)	4.5 (3.1, 5.7)	3.4 (2.3, 5.2)	ns	0.034	ns	ns
MoCA	19 (15, 21.5)	18.5 (14, 23)	24 (20, 26)	< 0.001	ns	< 0.001	< 0.001
MDS-UPDRS III	7 (4, 17.2)	0.5 (0, 3)	31.5 (16, 46)	< 0.001	< 0.001	< 0.001	< 0.001
PSP rating scale	12 (8.2, 15.8)	4 (2, 6)	31 (17, 42)	< 0.001	0.001	< 0.001	< 0.001
ASRS total v3	10 (5, 22.8)	0 (0, 1)	8 (3, 17.8)	< 0.001	< 0.001	ns	< 0.001
FAB	12 (8, 14.5)	15 (12.8, 17)	14 (12, 16)	0.006	0.003	0.004	ns
BNT	12 (9.5, 13)	3 (0, 6)	13 (12, 15)	< 0.001	< 0.001	0.002	< 0.001
Letter fluency sum	11 (3, 12)	19 (12, 30)	11 (6, 20)	0.001	0.001	0.048	0.007
WAB praxis	53.5 (44.2, 56.8)	59 (50.5, 60)	55 (49, 59)	0.007	0.006	ns	0.017
WAB AQ	79.3 (76.1, 88.3)	86.2 (68.2, 91.8)	96 (90.6, 97.8)	< 0.001	ns	< 0.001	< 0.001
WAB repetition	8.5 (7.6, 9)	9.1 (8.2, 9.6)	9.5 (9, 9.8)	< 0.001	0.031	< 0.001	0.018
WAB animal fluency	8 (4, 13)	7 (4, 10)	13 (10, 16.5)	< 0.001	ns	0.001	< 0.001
PPT word-word	48.5 (45.5, 50.2)	40 (33, 45)	49.5 (48, 51)	< 0.001	0.001	ns	< 0.001

Demographic and clinical data are reported as median (25th, 75th quartiles), number and percentage. *p*-values are from Kruskal–Wallis and Fisher’s exact tests. MoCA: Montreal cognitive assessment. MDS-UPDRS: movement disorder society-sponsored revision of the unified Parkinson’s disease rating scale. ASRS: apraxia of speech rating scale. FAB: frontal assessment battery. BNT: Boston naming test. WAB AQ: western aphasia battery aphasia quotient. PPT: pyramid and palm trees test. ns: not significant.

Cluster 2 (*n* = 44) mainly included svPPA (*n* = 25, 57%) and rtvFTD (*n* = 11, 25%) patients, with a small subset of PPA-other (*n* = 5, 11%), bvFTD (*n* = 2, 5%), and AOS-PAA (*n* = 1, 2%) patients (Figure 1). These patients had longer disease duration than the ones in cluster 1 (Table 2). They performed worse than the other two on BNT and PPT word-word. They also performed worse than cluster 3 on MoCA, WAB AQ, WAB repetition, and WAB animal fluency (Table 2). Gray matter volume loss was observed in the bilateral temporal lobes in these patients, particularly on the left side, and including the medial temporal lobe as well as in the anterior cingulum and orbitofrontal regions, compared to cognitively unimpaired individuals (Figure 2). When patients in cluster 2 were evaluated separately based on their clinical diagnoses, svPPA and rtvFTD patients showed bilateral temporal atrophy, with left- and right-sided emphasis, respectively (Figure 3). PPA-other patients showed exclusively left temporal volume loss, whereas the single AOS-PAA patient showed temporal volume loss, particularly in the right amygdala, as well as in the supplementary motor area and insula. Patients with bvFTD showed frontotemporal volume loss, which peaked in the right temporal pole (Figure 3).

Cluster 3 (*n* = 332) included all PSP (*n* = 185, 54%) and CBS (*n* = 23, 8%) patients, the majority of AOS-PAA and PPAOS (*n* = 106, 32%), some bvFTD (*n* = 6, 2%) and PPA-other (*n* = 9, 3%) patients, and a small subset of svPPA (*n* = 3, 1%) (Figure 1). The patients in this cluster were older than the ones in cluster 1 and 2 (Table 2). These patients were older than the ones in cluster 1 and 2 (Table 2). They performed worse than both cluster 1 and 2 on MDS-UPDRS III and PSP Rating Scale. They also performed worse than cluster 2 on ASRS, Letter Fluency Sum, WAB praxis (Table 2). Gray matter volume loss was predominantly subcortical, affecting the midbrain, basal ganglia, thalamus, and cerebellum, including the dentate nucleus, with cortical volume loss in the frontal lobe, extending into the parietal lobe (Figure 2). The PSP patients showed their characteristic pattern of gray matter volume loss, with key regions including the midbrain, dentate nucleus of the cerebellum, superior cerebellar peduncle, red nucleus, substantia nigra, thalamus, basal ganglia, and frontal cortex (Figure 3). The AOS-PAA, PPAOS, and CBS patients in cluster 3 showed volume loss in the thalamus, basal ganglia, and midbrain, as well as widespread cortical volume loss, including the supplementary motor area. PPA-other patients showed gray matter volume loss of the left supplementary motor area, midbrain and caudate. bvFTD patients showed volume loss in the left mid-frontal and left insula, as well as the thalamus and supplementary motor area. The three svPPA patients showed bilateral temporal volume loss, mostly restricted to the medial regions, primarily in the left hemisphere (Figure 3).

**Figure 4:**
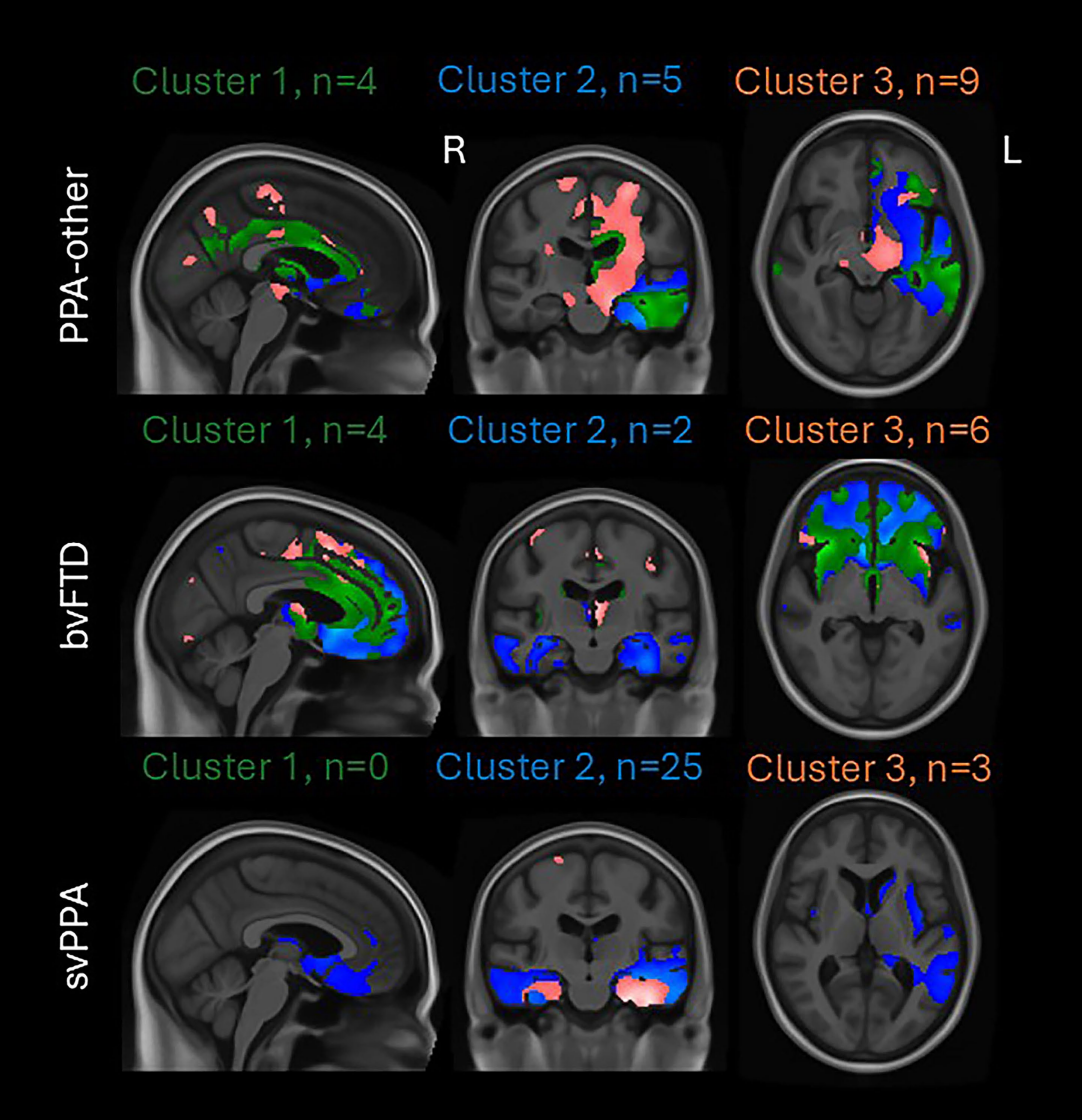
Atrophy patterns of PPA-other, svPPA, and bvFTD assigned to multiple clusters. SPM maps of atrophy (gray and white matter segmentation) in PPA-other (top row), bvFTD (middle row), and svPPA (bottom row) relative to cognitively unimpaired individuals shown at *p* < 0.001 without correcting for multiple comparisons, covarying for age, sex and scanner manufacturer. For each clinical diagnosis, the SPM maps of the patients in cluster 1, 2 and 3 are in green, blue and pink, respectively. The use of a more relaxed threshold helps capture the different atrophic patterns of patients with the same clinical diagnosis but assigned to different clusters.

Figure 4 shows the same maps of Figure 3 at a looser threshold (*p* < 0.001) to highlight how patients with the same clinical syndrome (PPA-other, svPPA or bvFTD) but assigned to different clusters showed different patterns of atrophy. Within the PPA-other group, the patients in cluster 1 showed left frontotemporal volume loss: two of them were classified as lvPPA and two as PAA. The PPA-other patients in cluster 2 showed more focal left temporal atrophy: two of them were classified as lvPPA, one as PAA, and the remaining two were unclassifiable PPA who progressed to svPPA at their follow-up visit. Finally, the PPA-other patients in cluster 3 showed involvement of the midbrain and supplementary motor area: seven of them were PAA and two were unclassifiable PPA (Figure 4). Within the bvFTD group, those in cluster 1 showed prefrontal volume loss, with greater temporal involvement in cluster 2 and thalamus and supplementary motor area involvement in cluster 3 (Figure 4). Within the svPPA group, the patients in clusters 2 and 3 both showed temporal lobe atrophy, although those in cluster 3 also showed precentral and supplementary motor area involvement while those in cluster 2 showed more involvement of the anterior cingulate and medial frontal lobe (Figure 4). Notably, all three svPPA patients assigned to cluster 3 were amyloid-positive on [^11^C] Pittsburgh Compound B PET. Supplementary Tables 1–5 contain the clinical and demographic characteristics of each syndrome within each cluster. PPAOS, AOS-PAA and PPA-other patients in cluster 1 were younger than the patients with the same clinical diagnosis in cluster 3 (Supplementary Tables 1–3). PPA-other and svPPA patients in cluster 2 were younger than the patients with the same clinical diagnosis in cluster 3 (Supplementary Tables 3, 4). AOS-PAA and PPA-other in cluster 1 performed worse on MOCA compared to cluster 3 (Supplementary Tables 2, 3). PPA-other patients in cluster 1 had a longer disease duration compared to cluster 3 (Supplementary Table 3). In cluster 1, PPAOS patients performed worse on WAB animal fluency (Supplementary Table 1); AOS-PAA patients on Letter fluency sum, WAB AQ and WAB praxis (Supplementary Table 2); and PPA-other patients on WAB repetition (Supplementary Table 3) compared to cluster 3. svPPA patients in cluster 2 performed worse on WAB AQ and better on MDS-UPDRS III compared to cluster 3 (Supplementary Table 4). bvFTD patients in cluster 1 performed better on the PSP rating scale compared to cluster 3 (Supplementary Table 5).

**Figure 5:**
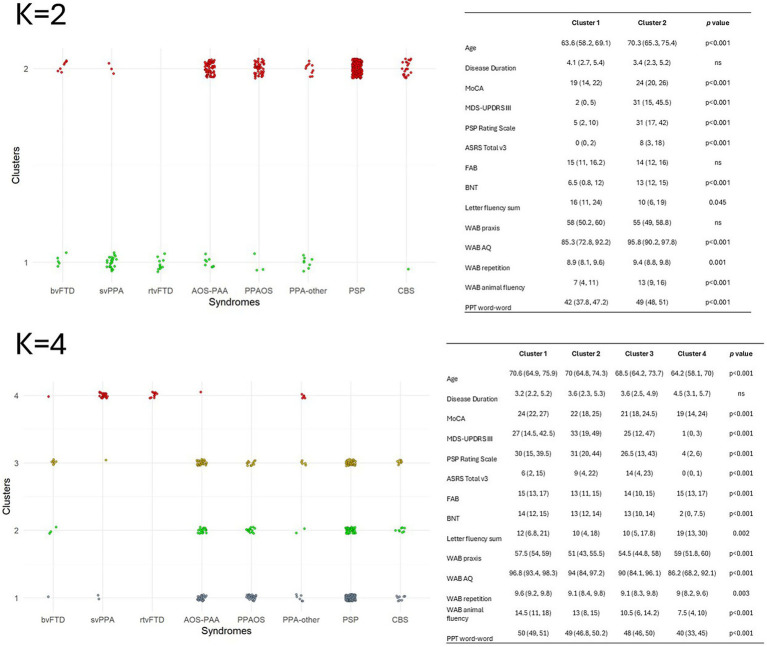
Syndrome distribution for *K* = 2 **(A)** and *K* = 4 **(B)** clustering. On the left, the assignment of the patients to the cluster solution with *K* = 2 or *K* = 4. On the right, the demographic and clinical characteristics of the patients within each cluster.

Secondary analysis with *K* = 2 and *K* = 4 is shown in Figure 5. In the *K* = 2 analysis, cluster 1 primarily captured language syndromes, while cluster 2 mainly captured atypical Parkinsonian syndromes. Patients in cluster 1 were younger, more cognitively impaired, performed worse on BNT, WAB AQ, WAB repetition, WAB animal fluency and PPT word-word and better on MDS-UPDRS III, PSP rating scale, ASRS and letter fluency sum than cluster 2. In the *K* = 4 analysis, the first three clusters primarily captured atypical Parkinsonian syndromes, where MoCA scores declined, and ASRS scores increased as one moved from cluster 1 to 3. Cluster 4 captured language syndromes and showed worse performance on MoCA, BNT, WAB AQ, and WAB animal fluency tests.

Clustering with *K* = 3 of principal components scores obtained from the raw gray and white matter segmentation maps of the FTD patients led to a CH index of 19.4 (Supplementary Figure 1). The three clusters were comparable to the ones obtained from *w*-scored images (Rand index: 0.91), pointing to the fact that syndromic heterogeneity was a larger source of variability in the data than covariates.

## 4 Discussion

This study used a structural MRI data-driven clustering approach to improve our understanding of how syndromic heterogeneity seen in FTD is reflected in structural neuroimaging on MRI. The cluster analysis split the cohort into three clusters with the first predominantly involving the frontal lobe, the second involving the temporal lobe, and the third involving midbrain, basal ganglia, and the frontal lobe. Most of the svPPA and rtvFTD patients were assigned to cluster 2 and most of the PPAOS patients and all the atypical Parkinsonian patients were assigned to cluster 3. However, the bvFTD, AOS-PAA, and PPA-other patients were split across clusters reflecting heterogeneity in patterns of volume loss within these syndromes.

The three-cluster analysis was used as the primary analysis for this study as it offered the most clinically meaningful separation of the FTD syndromes. The biggest axis of difference or highest cluster separation was observed with the two-cluster solution, which contrasted frontotemporal syndromes and atypical Parkinsonian syndromes (Murley et al., 2020). Instead, the four-cluster solution only provided further separation of the atypical Parkinsonian syndromes into smaller subsets.

Using the three-cluster approach, the first cluster was associated with frontal lobe volume loss and predominantly captured patients with bvFTD, AOS-PAA, and PPA-other, as well as three patients with PPAOS. This concurs with previous literature that shows that these syndromes affect the frontal lobes (Eldaief et al., 2023; Botha et al., 2015; Josephs et al., 2014a). Patients with bvFTD and PPA are also known to affect the temporal lobes (Eldaief et al., 2023; Gorno-Tempini et al., 2004) but the structural MRI-based clustering approach appears to have effectively captured cases of bvFTD and PPA-other with predominant frontal involvement.

Indeed, we have previously performed cluster analysis using MRI within patients with bvFTD and identified four anatomical subtypes: frontal-dominant, frontotemporal, temporal-dominant and temporo-parieto-frontal subtypes (Whitwell et al., 2009). The clinical profile of cluster 1 reflected the captured syndromes, with the presence of executive deficits, AOS and aphasia. Poor performance on executive functioning measures could be attributed to the presence of AOS-PAA (Polsinelli et al., 2022), PPA-other (Coemans et al., 2022), and bvFTD (Possin et al., 2013) in this cluster. Patients with AOS-PAA, PPAOS, and PPA-other patients show animal and letter fluency deficits (Scheffel et al., 2021; Tetzloff et al., 2025). There was also evidence of ideomotor apraxia and mild parkinsonism which may reflect the fact that the AOS-PAA and PPAOS patients often develop a parkinsonian disorder over time (Josephs et al., 2021), with features of PSP and CBS, and these patients commonly have a 4R tauopathy at autopsy (Spinelli et al., 2017; Seckin et al., 2020; Josephs and Duffy, 2008; Josephs et al., 2005).

The second cluster was associated with temporal lobe volume loss and as expected, captured all the rtvFTD and most of the svPPA patients. However, a couple of bvFTD patients were also captured in this cluster and these patients showed a frontotemporal pattern of volume loss. Several PPA-other patients were also captured in cluster 2 and these patients showed relatively focal involvement of the left temporal lobe. As expected from the high proportion of svPPA and rtvFTD patients, this cluster showed severe anomia and impairments in word knowledge (Hodges et al., 1992; Ulugut et al., 2024). Interestingly, bvFTD patients in this cluster tended to show worse performance on naming measures than those in the first cluster, possibly due to greater involvement of the temporal lobe, although the difference was not significant (Snowden, 2023; Rohrer, 2012).

The third cluster was associated with volume loss in brainstem, subcortical and frontal regions and captured all the PSP and CBS patients. This cluster was cognitively unimpaired, with parkinsonism and ideomotor apraxia observed on testing. Patients with PSP and CBS made up 63% of this cluster, and the presence of parkinsonism and ideomotor apraxia is consistent with the characteristic features of PSP (Höglinger et al., 2017) and CBS (Armstrong et al., 2013). This cluster also included nearly all the PPAOS patients and most of the AOS-PAA patients. As discussed above, patients with PPAOS and AOS-PAA commonly develop the clinical features of PSP and CBS, as well as midbrain atrophy, over time (Whitwell et al., 2019). PPAOS patients can overlap anatomically with PSP (Whitwell et al., 2013). The AOS-PAA patients captured in cluster 3 showed more atrophy in the brainstem and subcortical structures than the AOS-PAA patients in cluster 1, suggesting they may be more advanced in their disease course. Indeed, the AOS-PAA patients in cluster 3 showed a non-significant trend for worse parkinsonism and significantly higher cognitive impairment than those in cluster 1. However, patients with svPPA, bvFTD, and PPA-other were also captured in the third cluster. The bvFTD patients in cluster 3 did show evidence of parkinsonism and the pattern of volume loss focally affected the supplementary motor area which may explain why they clustered with the AOS-PAA and PPAOS who show involvement of the supplementary motor area (Josephs et al., 2021). Patients with bvFTD can have PSP pathology (Hassan et al., 2012) and parkinsonian features (Park et al., 2017). The PPA-other patients in cluster 3 tended to show greater involvement of subcortical structures. It is not surprising that the clinical heterogeneity of the PPA-other group was mirrored by heterogeneity in the patterns of atrophy, which was captured by the clustering algorithm. It is less clear why three svPPA patients were captured in cluster 3 since these patients still showed predominant temporal lobe volume loss, although there was some subtle involvement of the supplementary motor area. These three patients were amyloid-positive on PET and could possibly have Alzheimer’s disease (AD) pathology, but it is also possible that in these patients FTLD coexists with AD, which has been reported previously (Santos-Santos et al., 2018). Furthermore, patients with svPPA rarely report AD pathology (Spinelli et al., 2017; Harris et al., 2013; Davies et al., 2005; Mesulam et al., 2014), which makes it unlikely that all three patients will have AD pathology at autopsy. More importantly, these three svPPA patients in cluster 3 were older than the rest of the svPPA cohort suggesting that the older age (Dickson et al., 1992; Rodrigue et al., 2009) could be a more likely explanation for the presence of amyloid deposition.

The strengths of this study include a well-characterized large cohort of FTD clinical syndromes, with consistent neuroimaging protocols and extensive clinical evaluations. One potential limitation of this study is a skewed cohort with a large number of AOS-PAA, PPAOS, and PSP patients, due to the nature of the interests of our laboratory. Another limitation is the absence of neuropathological and genetic data, which limits the biological interpretability of the identified clusters. Although our ensemble clustering approach with hierarchical algorithms led to a clinically meaningful grouping of syndromes, it is possible that other clustering and more advanced machine learning approaches would lead to different groupings of structural MRI features. The clusters reported in our study were obtained employing PCA to discover the imaging components explaining the most variance across FTD patients. The linearity assumption of PCA may not always hold true in the relationship among brain structures and could not properly account for scanner manufacturer variability, which represent other limitations of our study.

## 5 Conclusion

Our findings highlight the heterogeneity in FTD, which can be captured by three main patterns on structural MRI: frontal, temporal and subcortical. These findings also bring to light the existence of heterogeneity within a syndrome as we observed more than one anatomical subtype in PPAOS, AOS-PAA, svPPA, and bvFTD, implying that the pathophysiological mechanisms underlying these anatomical patterns may be complex and heterogeneous. Together these findings add to our current knowledge of FTD syndromes and lay the foundation for future research in personalized medicine that could guide therapeutic decisions in patients with these syndromes. They also highlight that a dimensional approach would be beneficial in personalized treatment decisions and clinical trials that use atrophy patterns as outcome measures.

## Data Availability

The raw data supporting the conclusions of this article will be made available by the authors, without undue reservation.

## Footnotes

1
https://www.nitrc.org/projects/mcalt/

## Glossary

FTD: frontotemporal dementia
bvFTD: behavioral variant of FTD
rtvFTD: right temporal variant of FTD
svPPA: semantic variant of primary progressive aphasia
PAA: progressive agrammatic aphasia
AOS: apraxia of speech
PPAOS: primary progressive AOS
PSP: progressive supranuclear palsy
CBS: corticobasal syndrome
MoCA: Montreal cognitive assessment
MRI: magnetic resonance imaging
MPRAGE: magnetization prepared rapid gradient echo
NRG: neurodegenerative research group
PET: positron emission tomography
BNT-SF: 15-item Boston naming test-short form
PPT: pyramids and palm trees
MDS-UPDRS III: movement disorders society sponsored revision of the unified Parkinson’s disease rating scale III
WAB: western aphasia battery
FAB: frontal assessment battery
WAB AQ: WAB aphasia quotient
ASRS: apraxia of speech rating scale
TIV: total intracranial volume
