# Supplementary material for: Frontotemporal dementia: does structural MRI-based clustering match clinical syndromes?
Source: Front Neurosci. 2026 May 5;20:1771092. doi: 10.3389/fnins.2026.1771092 (PMC13184375; doi:10.3389/fnins.2026.1771092)
Supplement: Supplementary file 1 [file Data_Sheet_1.DOCX]

**Supplemental Figure 1.** Syndrome distribution for K=3 clustering applied on principal components scores obtained from the raw grey and white matter segmentation maps of the FTD patients, without *w*-scoring.

**
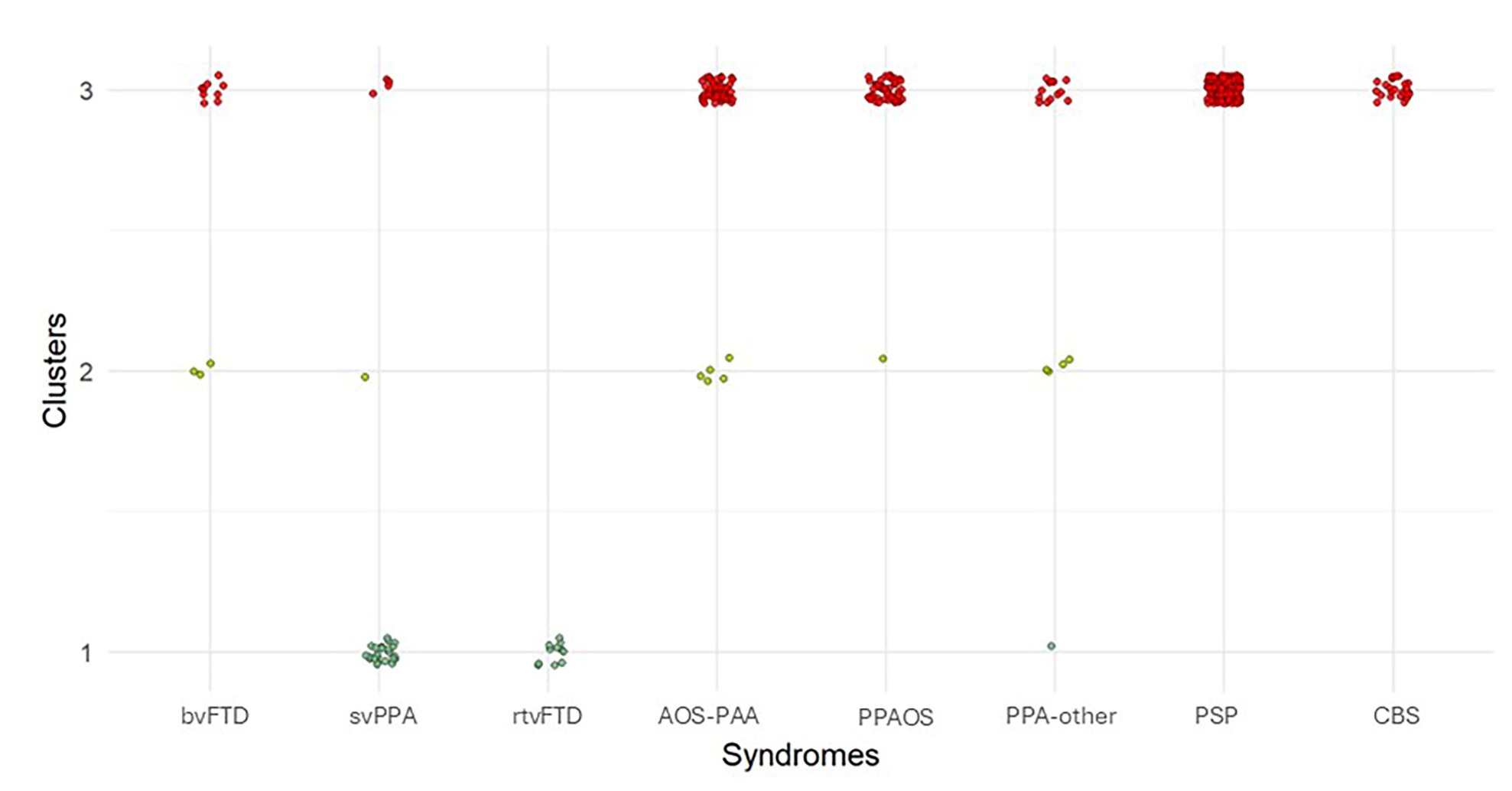
**

**Supp Tables 1-5.** Demographic and clinical characteristics of the participants of each syndrome within each cluster. Only syndromes whose participants were present in more than one cluster are reported. ns: not significant. NA: not available.

| **PPAOS** | | | | |
| --- | --- | --- | --- | --- |
|  | **Cluster 1**  **N=3** | **Cluster 2**  **N=0** | **Cluster 3**  **N=50** | **p value**  **cluster 1-3** |
| **Age** | 59.7 (51.7, 60.5) | - | 72.8 (64.6, 77.5) | 0.017 |
| **Disease Duration** | 3.4 (2.2, 3.6) | - | 3.3 (2.1, 5.4) | ns |
| **MoCA** | 22 (19, 24.5) | - | 27 (25, 28) | ns |
| **MDS-UPDRS III** | 11 (6, 11) | - | 12 (5, 22) | ns |
| **PSP Rating Scale** | 7 (4.5, 9.5) | - | 8 (4, 17) | ns |
| **ASRS Total v3** | 20 (13, 22.5) | - | 15.5 (11.8, 22.5) | ns |
| **FAB** | 15 (10.5, 15.5) | - | 17 (15.8, 17) | ns |
| **BNT** | 13 (12.5, 13.5) | - | 15 (13, 15) | ns |
| **Letter fluency sum** | 9 (5.5, 12.5) | - | 22 (13.5, 31.8) | ns |
| **WAB praxis** | 58 (57.5, 59) | - | 58 (55, 59) | ns |
| **WAB AQ** | 96.2 (86.2, 96.8) | - | 97.8 (96.8, 99.2) | ns |
| **WAB repetition** | 9.3 (9.1, 9.6) | - | 9.7 (9.4, 9.9) | ns |
| **WAB animal fluency** | 8.5 (6.2, 10.8) | - | 16 (15, 20) | 0.047 |
| **PPT word-word** | 51 (51, 51) | - | 50 (49, 51) | ns |

| **AOS-PAA** | | | | |
| --- | --- | --- | --- | --- |
|  | **Cluster 1**  **N=13** | **Cluster 2**  **N=1** | **Cluster 3**  **N=56** | **p value**  **cluster 1-3** |
| Age | 65.6 (57.9, 70.1) | 63 | 70.4 (65.2, 74.4) | 0.017 |
| Disease Duration | 2.9 (2.6, 5.3) | 9.3 | 4 (2.6, 5.7) | ns |
| MoCA | 17 (14, 21) | 16 | 23 (19, 25) | 0.008 |
| MDS-UPDRS III | 12 (6, 19) | 17 | 17 (10.5, 27) | ns |
| PSP Rating Scale | 13 (11, 20) | 15 | 20 (10, 29) | ns |
| ASRS Total v3 | 20 (10, 25) | NA | 23 (13.2, 29.8) | ns |
| FAB | 12 (8, 14) | 3 | 14 (10, 16) | ns |
| BNT | 12 (9.8, 13) | 6 | 12.5 (11, 14) | ns |
| Letter fluency sum | 6 (2.5, 11.5) | 4 | 11 (7, 19) | 0.04 |
| WAB praxis | 49 (39, 53) | 40 | 54 (46, 58) | 0.03 |
| WAB AQ | 77.7 (75.6, 85.5) | NA | 88 (81.4, 93.4) | 0.019 |
| WAB repetition | 8.1 (7.6, 8.9) | NA | 9 (7.8, 9.6) | ns |
| WAB animal fluency | 8 (5.5, 13) | 7 | 11 (8, 14.8) | ns |
| PPT word-word | 48.5 (42.8, 49) | NA | 49 (48, 50.5) | ns |

| **PPA-other** | | | | |  |  |
| --- | --- | --- | --- | --- | --- | --- |
|  | **Cluster 1**  **N=4** | **Cluster 2**  **N=5** | **Cluster 3**  **N=9** | **p value**  **cluster 1 2** | **p value**  **cluster 1 3** | **p value**  **cluster 2 3** |
| **Age** | 64.9 (60.9, 66.3) | 58.2 (58, 62.3) | 69.5 (66.1, 77.3) | ns | 0.045 | 0.02 |
| **Disease Duration** | 3.5 (2.8, 4.4) | 3.2 (2.1, 5.7) | 2 (1.2, 2.1) | ns | 0.005 | ns |
| **MoCA** | 18 (17.5, 19) | 22 (18, 23) | 24 (21, 25) | ns | 0.026 | ns |
| **MDS-UPDRS III** | 4 (3.2, 5) | 1 (0, 2) | 4 (2, 9) | ns | ns | ns |
| **PSP Rating Scale** | 15 (15, 15) | 6 (6, 6) | 3 (1, 9) | ns | ns | ns |
| **ASRS Total v3** | 0 (0, 0.5) | 1.5 (0, 3) | 2 (1, 4) | ns | ns | ns |
| **FAB** | 13 (9.5, 13.5) | 16 (10, 17) | 15 (11, 16) | ns | ns | ns |
| **BNT** | 6.5 (1.5, 11.2) | 7 (4.5, 9.5) | 10 (9.5, 14) | ns | ns | ns |
| **Letter fluency sum** | 14.5 (12.8, 16.2) | 32 (18.5, 38.5) | 10 (6, 22) | ns | ns | ns |
| **WAB praxis** | 57 (55.5, 57) | 57 (51.2, 59) | 55 (53, 58) | ns | ns | ns |
| **WAB AQ** | 79.3 (72.9, 82.3) | 87.6 (61.3, 92.8) | 90.4 (89.3, 92.7) | ns | ns | ns |
| **WAB repetition** | 8.2 (7.4, 8.5) | 8.2 (7.4, 9.8) | 9.4 (9.1, 9.6) | ns | 0.041 | ns |
| **WAB animal fluency** | 7 (4, 11.5) | 8 (4, 18) | 10 (8, 11) | ns | ns | ns |
| **PPT word-word** | 48 (44, 49.5) | 48 (47, 49) | 49 (46, 51) | ns | ns | ns |

| **svPPA** | | | | |
| --- | --- | --- | --- | --- |
|  | **Cluster 1**  **N=0** | **Cluster 2**  **N=25** | **Cluster 3**  **N=3** | **p value**  **cluster 2 3** |
| **Age** | - | 67.6 (58.6, 70.5) | 78.4 (73.4, 79.8) | 0.049 |
| **Disease Duration** | - | 4.5 (3.1, 5.7) | 1.9 (1.5, 4.5) | ns |
| **MoCA** | - | 18 (13.5, 21.2) | 21 (20, 22) | ns |
| **MDS-UPDRS III** | - | 0 (0, 2) | 5 (3.5, 7) | 0.021 |
| **PSP Rating Scale** | - | 4.5 (3, 7.2) | 5 (3.5, 5.5) | ns |
| **ASRS Total v3** | - | 0 (0, 1) | 1 (0.5, 1) | ns |
| **FAB** | - | 15 (12, 17) | 16 (15.5, 16) | ns |
| **BNT** | - | 0 (0, 2) | NA | ns |
| **Letter fluency sum** | - | 16 (12, 27) | 19 (18.5, 23.5) | ns |
| **WAB praxis** | - | 56 (45, 59) | 59 (58, 59.5) | ns |
| **WAB AQ** | - | 79.2 (63.2, 87.7) | 95.8 (95.2, 95.9) | 0.005 |
| **WAB repetition** | - | 8.8 (7.4, 9.2) | 9.2 (9.2, 9.4) | ns |
| **WAB animal fluency** | - | 6.5 (2, 9) | 9 (8.5, 12) | ns |
| **PPT word-word** | - | 38.5 (30.8, 43.2) | 42 (38.5, 44) | ns |

| **bvFTD** | | | | |  |  |
| --- | --- | --- | --- | --- | --- | --- |
|  | **Cluster 1**  **N=4** | **Cluster 2**  **N=2** | **Cluster 3**  **N=6** | **p value**  **cluster 1 2** | **p value**  **cluster 1 3** | **p value**  **cluster 2 3** |
| **Age** | 61.7 (55.7, 66.3) | 61.3 (58.7, 63.8) | 73.2 (67.3, 74.5) | ns | ns | ns |
| **Disease Duration** | 3.3 (2.3, 4.1) | 3.4 (3.4, 3.4) | 3.7 (2.2, 4) | ns | ns | ns |
| **MoCA** | 20.5 (18, 22) | 15 (14.5, 15.5) | 16 (15, 23) | ns | ns | ns |
| **MDS-UPDRS III** | 2 (0, 12.2) | 2 (1, 3) | 24 (12, 45) | ns | ns | ns |
| **PSP Rating Scale** | 3 (2.2, 6) | 6 (6, 6) | 22 (13, 23) | ns | 0.049 | ns |
| **ASRS Total v3** | 2 (2, 4) | 2 (1, 3) | 6 (6, 10) | ns | ns | ns |
| **FAB** | 12 (9.5, 14.5) | 11.5 (9.8, 13.2) | 13 (5.5, 13.8) | ns | ns | ns |
| **BNT** | 12 (12, 12) | 4 (4, 4) | 12 (10, 12.5) | ns | ns | ns |
| **Letter fluency sum** | 11 (9, 11.2) | 11 (11, 11) | 10.5 (9.8, 11.2) | ns | ns | ns |
| **WAB praxis** | 56 (50, 58) | 59 (58.5, 59.5) | 60 (43, 60) | ns | ns | ns |
| **WAB AQ** | 93.3 (77.2, 94.9) | 90.7 (90.5, 90.8) | 87.6 (86.9, 90.2) | ns | ns | ns |
| **WAB repetition** | 9 (7.2, 9.4) | 9.6 (9.4, 9.8) | 9.3 (8.8, 9.5) | ns | ns | ns |
| **WAB animal fluency** | 10 (7, 13.5) | 11.5 (11.2, 11.8) | 9.5 (8.5, 11.5) | ns | ns | ns |
| **PPT word-word** | 49 (48, 50) | 45 (43.5, 46.5) | 47.5 (46.8, 48.2) | ns | ns | ns |
